# Physiology of bridging stent grafts after fenestrated/branched endovascular aortic repair: Where translational science meets the clinical profile

**DOI:** 10.1113/EP091813

**Published:** 2025-01-27

**Authors:** Thurkga Moothathamby, Matti Jubouri, Tharun Rajasekar, Subham Roy, Maya Alfwaress, Samuel S. S. Rezk, Samuel N. S. Ghattas, Mario D'Oria, Damian M. Bailey, Ian M. Williams, Mohamad Bashir

**Affiliations:** ^1^ Barts and The London School of Medicine and Dentistry Queen Mary University of London London UK; ^2^ Hull York Medical School University of York York UK; ^3^ Liverpool Medical School University of Liverpool Liverpool UK; ^4^ Faculty of Medicine Jordan University of Science and Technology Irbid Jordan; ^5^ Department of General Surgery Ysbyty Glan Clwyd Rhyl UK; ^6^ Division of Vascular and Endovascular Surgery, Department of Medical, Surgical and Health Sciences University of Trieste Trieste Italy; ^7^ Neurovascular Research Laboratory, Faculty of Life Sciences and Education University of South Wales Pontypridd UK; ^8^ Department of Vascular Surgery University Hospital of Wales Cardiff UK

**Keywords:** balloon‐expandable, branched, bridging stent graft, complex endovascular aortic repair endovascular aortic repair, endovascular aortic repair, fenestrated, haemorheology, self‐expanding, vessel physiology

## Abstract

Fenestrated/branched endovascular aortic repair emerges as the primary therapeutic modality for intricate aortic pathologies encompassing the paravisceral and thoracoabdominal segments, where bridging stent grafts (BSGs) play a vital role in linking the primary aortic endograft with target vessels. Bridging stent grafts can be categorized mainly into self‐expanding stent grafts (SESGs) and balloon‐expandable stent grafts (BESGs). Physiological factors significantly influence post‐complex endovascular aortic repair BSG behaviour, impacting clinical outcomes of SESGs and BESGs in different but overlapping ways. Crucial prerequisites for BSGs encompass not only flexibility but also resilience against mechanical stress and compliance mismatch, especially when bridging the rigid aortic main body with dynamic target vessels. The significance of considering these physiological factors in clinical decision‐making is underscored by recognizing the interplay between SESG and BESG characteristics, vessel physiology and patient haemorheology. Such factors include the anatomy and tortuosity of the vessel, diameter of the vessel and BSG, deployment and durability, extrinsic stenosis and respiratory motion. Haemorheological factors, such as anti‐thrombotic therapy and hydration status, need to be considered. This narrative review examines both in vitro and in vivo evidence regarding the impact of physiological factors on the behaviour of BSGs and assesses the consequences for clinical outcomes following complex endovascular aortic repair.

## Abstract (highlights)

- **What is the topic of this review?**
  The narrative review aims to examine both in vitro and in vivo evidence regarding the impact of physiological factors on the behaviour of bridging stent grafts (BSGs) and to assess the consequences for clinical outcomes following fenestrated/branched endovascular aortic repair (F/BEVAR).
- **What advances does it highlight?**
  Bridging stent grafts play a major role in F/BEVAR. Physiological factors significantly influence post‐F/BEVAR BSG behaviour, impacting clinical outcomes. The significance of considering these physiological factors in clinical decision‐making is underscored by recognizing the interplay between BSG characteristics, vessel physiology and patient haemorheology.

## 1 INTRODUCTION

Fenestrated/branched endovascular aortic repair (F/BEVAR), commonly referred to as complex EVAR, emerges as the primary therapeutic modality for intricate aortic pathologies encompassing the paravisceral and thoracoabdominal segments, demonstrating both safety and efficacy in the management of juxta‐renal and supra‐renal abdominal aortic aneurysms (AAAs) and thoracoabdominal aneurysms. This has been shown in some studies to result in similar short‐ and long‐term mortality and morbidity to open surgical repair (Zlatanovic et al., 2023). Nevertheless, the endovascular approach is considered the foremost intervention owing to its efficacy in addressing complex aortic conditions, as also endorsed by contemporary clinical practice guidelines (Wanhainen et al., 2024). The successful execution of complex EVAR demands a high level of expertise and specialized training in endovascular techniques. Typically conducted by interventional radiologists or vascular surgeons with specific experience in complex endovascular procedures, these practitioners navigate the intricacies of creating customized stent grafts to address complex aortic pathologies. The critical aspect of patient selection involves a comprehensive assessment of overall health, anatomical considerations and suitability for this advanced endovascular approach; once the procedure is completed, ongoing patient care is paramount. Regular and thorough follow‐up is essential for monitoring the performance of the stent graft, early detection of potential complications and ensuring the long‐term well‐being of the patient (Chait et al., 2022). This comprehensive approach, from specialized training to meticulous patient selection and vigilant postoperative care, underscores the nuanced nature of complex EVAR as a highly advanced and patient‐centric intervention in vascular surgery.

**FIGURE 1:**
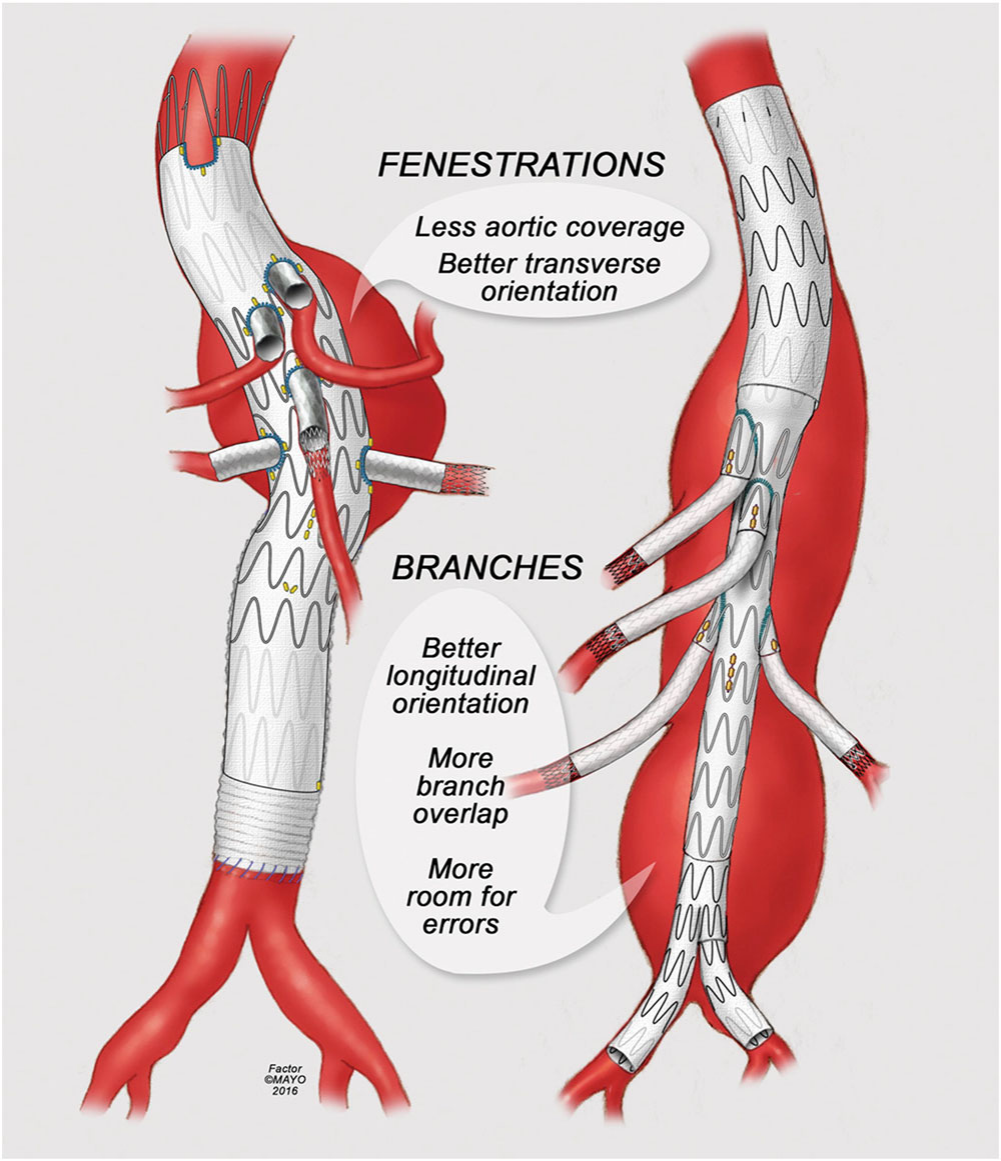
Visual illustration of fenestrated (left) and branched (right) endovascular aortic repair. Re‐used from Oderich et al. (2017), with permission.

Bridging stent grafts (BSGs) play a vital role in linking the primary aortic endograft with target vessels (TVs), particularly those involving complex anatomies or vital branch vessels, necessitating the provision of enduring and advantageous perfusion to the TV alongside the establishment of a proper component seal (Yoon et al., 2021) (Figure 1). Bridging stent grafts are indicated for conditions such as thoracoabdominal and pararenal aneurysms, where these stents are used in minimally invasive procedures to preserve blood flow to essential arteries whilst excluding the aneurysm sac. In general, BSGs are available in several types with different characteristics; yet, the most commonly used versions include self‐expanding stent grafts (SESG) and balloon‐expandable stent grafts (BESG). Although there are no current guidelines with regard to the appropriate type of BSG to use, BESGs are preferred in FEVAR owing to their rigidity and the ability to flare. In contrast, SESGs provide flexibility, which is desirable for tortuous vessels that often require branched grafts during BEVAR (Tenorio et al., 2020). Bridging stent grafts can also be categorized into covered stents and bare metal stents (BMSs), with the literature suggesting that covered stents provide more favourable outcomes owing to a lower rate of restenosis and fractures (BD, 2023; Bekken et al., 2022). There is a wide range of balloon‐expandable and self‐expanding stents available commercially, most of which are covered. However, none of these has been licenced specifically for use as a BSG in complex EVAR and is being used off the shelf (Bryce et al., 2018).

The use of BSGs is not without potential complications, including endoleaks, kinks, fractures, migrations, occlusions, stenosis and perforations (Torsello, Herten, Müller, et al., 2020). Caution is warranted in patient selection to ensure the optimal outcomes and minimize these risks. Hence, it is important to draw out aspects such as types of BSGs, anatomy and physiology of the vessels and haemorheology of the patient to understand pathways for optimal implantation and subsequent aortic remodelling. Physiological factors significantly influence post‐F/BEVAR BSG behaviour, impacting clinical outcomes with complications. Crucial prerequisites for BSGs encompass not only flexibility but also resilience against mechanical stress and compliance mismatch, especially when bridging the rigid aortic main body with dynamic TVs. The significance of considering physiological factors in clinical decision‐making is underscored by recognizing the interplay between BSG characteristics, vessel physiology and patient haemorheology. Overall, this narrative review focuses on examining both in vitro and in vivo evidence regarding the impact of physiological factors on the behaviour of BSGs and assessing the consequences for clinical outcomes, such as endoleaks and stent fractures.

## 2 SELF‐EXPANDING BRIDGING STENT GRAFTS VERSUS BALLON‐EXPANDABLE BRIDGING STENT GRAFTS

Self‐expanding BSGs are used for a variety of different vessels, including the coeliac, mesenteric, iliac, popliteal, axillary, subclavian and sciatic arteries. They can be divided into two mechanical subtypes, these being spring mechanism and the more novel method, which involves temperature‐dependent expansion of the metallic graft using the memory of the materials (Xue et al., 2020). This mechanism is used in stents made of nitinol. After implantation of the stent, an outward force is applied in a slow and controlled manner after positioning against the vessel wall. With regard to the delivery system of the stents, this involves the utilization of inner and outer catheters, which are locked together. Owing to their flexibility, they have been preferred for directional branches (Yoon et al., 2021). One documented drawback of SESGs is the tendency for the leading end to move forwards, which leads to some degree of stent instability (Hamer et al., 2004). In addition, the literatures points towards a higher risk of debris being released from embolisms because of the free‐cell area coverage of the SESGs (Ellenbogen et al., 2017). A summary of the main SESGs available commercially can be found in Table 1 (Gennai et al., 2019, 2022; Gore & Associates, 2016; Mafeld et al., 2019; Rzucidlo et al., 2003; Tielliu et al., 2005; Xenos et al., 2003).

**TABLE 1:** Summary of the main commercially available self‐expanding bridging stent grafts.

Model	F/BEVAR	Target vessel	Diameter (mm)
Fluency plus flair	Branched	Coeliac and mesenteric	6–13.5
VBX	Branched	Superficial femoral, iliac, popliteal	5–16
Covera	Branched	Iliac, sciatic	6–10
Wallgraft	Branched	Aorto‐iliac, popliteal, subclavian, axillary	6–14

Abbreviations: F/BEVAR, fenestrated/branched endovascular aortic repair; VBX, Viabahn balloon‐expandable stent.

In contrast, BESGs have a wide range of indications, including alleviating atherosclerotic and occlusive disease within the aorto‐iliac, mesenteric, femoropopliteal, renal, vertebral and cerebral arteries. The stents are usually made from stainless steel or cobalt chromium and come in lengths of 15–79 mm (Yoon et al., 2021). They are designed with a crimped orientation and placed over a balloon‐tipped catheter. Once the catheter has reached the target location within the artery, stent expansion is facilitated by inflation of the balloon, which is deflated and removed afterwards (Vishnu et al., 2022). In a prospective multicentred observational study, Usai et al. (2023) noted a high incidence of graft occlusions within renal vessels, pointing out a potential area of caution when treating lesions within these vessels. This is largely the result of their tortuous and diverse anatomical structures (Usai et al., 2023). Some studies have also highlighted the higher rates of target visceral vessel instability with BESGs. Like SESGs, several BESG options are available on the market, a summary of which can be found in Table 2 (FDA, 2017; Abbott, 2023; Katsargyris et al., 2023; Mwipatayi et al., 2020; Sayed et al., 2019; Spear et al., 2018; Torsello et al., 2020; van der Riet et al., 2021).

**TABLE 2:** Summary of the main commercially available balloon‐expanding bridging stent grafts.

Model	F/BEVAR	Target vessel	Diameter (mm)
Advanta V12/iCast	Fenestrated	Mesenteric, brachiocephalic, aorto‐iliac and small renal vessels	5–10
BeGraft and BeGraft plus	Fenestrated	Renal	5–10
E‐ventus	Fenestrated	Juxtarenal and common iliac	5–10
LifeStream	Fenestrated	Iliac	5–10
Abbot Vascular GraftMaster RX	Fenestrated	Coronary	2.8–4.8
VBX	Fenestrated/Branched	Iliac and renal	5–16

Abbreviations: F/BEVAR, fenestrated/branched endovascular aortic repair; VBX, Viabahn balloon‐expandable stent.

Consequently, when implanting bridging stents into tortuous anatomy, it is essential to use flexible stents. Self‐expanding BSGs are typically more adaptable than BESGs and are therefore preferred for directional branches, because they can accommodate various angles within the target vessel (Kim et al., 2022). Additionally, extending the distal end of the covered stent with a self‐expandable BMS might be necessary to prevent kinks or abrupt angulation in a TV following complex EVAR. Table 3 provides a comparative overview of the main features, indications, risks, advantages and disadvantages of SESGs and BESGs.

**TABLE 3:** Summary of comparison between self‐expanding stent grafts and balloon‐expandable stent grafts.

Characteristic	Self‐expanding stent grafts	Balloon‐expandable stent grafts
Materials	Typically made from nitinol (nickel–titanium alloy)	Made from stainless steel or cobalt chromium
Expansion mechanism	Temperature‐dependent or spring‐based expansion	Expanded by inflating a balloon
Deployment process	Inner and outer catheters locked together; slow and controlled outward force	Crimped on a balloon‐tipped catheter; expanded via balloon inflation
Indications	Used for various vessels: coeliac, mesenteric, iliac, popliteal, axillary, subclavian and sciatic arteries	Indicated for atherosclerotic and occlusive diseases in aorto‐iliac, mesenteric, femoropopliteal, renal, vertebral and cerebral arteries
Adaptability	More adaptable, preferred for tortuous and directional branches	Less adaptable, less suitable for tortuous anatomy
Stent migration risk	Higher risk of stent instability and forward movement of the leading end	Less prone to migration but higher risk of graft occlusions
Embolism risk	Higher risk of debris release owing to free‐cell area coverage	Moderate embolism risk, although no explicit mechanism noted
Advantages	Flexible for directional branches Adapts well to tortuous anatomy	Memory‐controlled and precise deployment Effective in shorter, more straightforward vessel segments
Disadvantages	Instability at the leading end Potential for formation of debris and emboli	Higher risk of occlusions in tortuous vessels, such as the renal arteries Stiffer, less flexible in tortuous anatomy
Clinical outcomes	Studies show conflicting outcomes regarding branch failure and patency rates	Studies show higher occlusion in vessels with complex anatomy

Overall, studies by Tenorio et al. (2020) and Mastracci et al. (2016) present conflicting results regarding the outcomes of SESGs and BESGs as bridging devices in branched thoracoabdominal aneurysm repair. A multivariate analysis showed no difference between the two (*P* = 0.91), with the renal branch (vs. visceral branch, *P* = 0.001) being the only variable associated with branch failure (Mastracci et al., 2016), and the authors found no significant difference in branch failure. Tenorio et al. (2020) reported superior primary patency in SESGs. These discrepancies underscore the need for continuous investigation, emphasizing the importance of collecting more data on new BSGs.

## 3 PHYSIOLOGY OF BRIDGING STENT GRAFTS

In this section, we explore key factors influencing the performance of SESGs and BESGs in complex EVAR. Crucial elements, such as vessel anatomy, diameter, tortuosity, stent sizing and radial force, are discussed alongside deployment challenges, complications of extrinsic stenosis and the role of covered stents. Consideration is also given to the effects of respiratory motion, cyclical stress, haemodynamics and long‐term durability of the BSGs. By examining the similarities and differences between SESGs and BESGs, this review highlights how each is optimized for clinical success in complex aortic interventions.

### 3.1 Anatomy and diameter of vessel

Both SESGs and BESGs present distinct advantages and limitations based on their interaction with vessel geometry. Renal arteries, for instance, warrant particular attention, presenting higher TV event rates than mesenteric vessels (6%–8% vs. 2%–3%) (Kim et al., 2022; Mastracci et al., 2016). The specific aetiologies for increased reintervention rates in renal targets remain unclear, with proposed hypotheses including smaller vessel diameter, the presence of ostial stenosis, variable renal angle, and respiratory motion (Kim et al., 2022). The initial clinical experience of Toresello et al. (2020) underscores the importance of maximizing the landing zone in the TV. Attempts to preserve polar arteries in early renal artery branching might elevate the risk of type IC endoleaks. Furthermore, the orientation of renal arteries in BEVAR appears to impact stent durability significantly. Gallitto et al. (2018) identified that both ascending and descending‐followed‐by‐ascending orientations of the renal artery were independently correlated with composite TV events, presenting 4‐ and 11‐times higher risks of renal artery loss, respectively. Additionally, the placement of renal stents has the potential to modify the angle or curvature of the native renal artery beyond the stent, thereby influencing its long‐term patency (Kim et al., 2022; Ullery et al., 2016). Branched endografts pose greater risks of TV loss (9% vs. 2%, *P* = 0.0001) (Hamer et al., 2004). As more long‐term data on complex EVAR stent graft durability become available, it will guide the selection of bridging stents and incorporation techniques for visceral vessels.

In contrast, BESGs have traditionally been used for transversely oriented vessels aligned by fenestrations, owing to their rigidity, shorter length availability, and the capacity to flare (Ullery et al., 2016). This rigidity and fenestrated design are particularly well suited for addressing complex juxta‐renal or supra‐renal AAAs, where precise placement is crucial. Given that TVs can exhibit conical or fusiform morphologies, the use of BESGs necessitates careful adaptation to the vessel wall through moderate sizing. A BESG such as the Viabahn balloon‐expandable stent (VBX) stands out in this context, because it can be dilated up to 16 mm (Table 2). Preliminary experience indicates that its primary patency at 24 months is comparable to that of the Fluency stent from Bard Medical (98.1% vs. 98.6%; *P* = 0.095) (Motta et al., 2021; BD, 2016). This attribute might potentially address challenges associated with bridging large vessels in branched grafts (Kim et al., 2022). Early observations from the experience of Mastracci et al. (2016) highlight that the VBX stent offers superior stent circularity in comparison to other devices, despite some shortening being noted. This emphasizes the importance of carefully deciding on the nominal stent length (Finotello et al., 2021). However, it is important to note that long‐term durability data on VBX stents have not yet been reported. Likewise, newer low‐profile stents, such as the BeGraft, have more recently been used in clinical practice with satisfactory outcomes up to 2 years (D'Oria, Mezzetto, Silingardi, et al., 2022). Their low profile might enable easier implantation using smaller sheaths; however, how they will behave over several cycles of superimposed mechanical stress and at longer‐term follow‐up remains an area of active research (D'Oria, Mezzetto, Silingardi, et al., 2022).

Interestingly, visceral vessel patency appears less influenced using directional branches than renal arteries. These vessels tend to have downward‐curving geometries, which are generally less prone to the acute angles seen in renal arteries. In a study involving 335 renal‐mesenteric arteries targeted by directional branches, one of the factors independently associated with the loss of primary patency was the specific renal artery target (Oderich et al., 2011). Experts suggest that directional branches might offer advantages in bridging the steep downward angles (Grimme et al., 2014) of coeliac and superior mesenteric arteries, because these arteries typically course caudally, whereas renal arteries often traverse laterally or cranially (Troisi et al., 2011; Yoon et al., 2021). Directional cuffs could also be more beneficial in cases of a large aneurysmal aorta, allowing for adaptation to the reduction in aneurysmal diameter over time and spanning the significant gap between the stent graft and the vessel origin (Oderich, Ribeiro, Hofer, et al., 2017). It has been observed that when a considerable distance exists between the aortic device and the origin of the TV (>5 mm), there is an elevated risk of target vessel instability (Troisi et al., 2011). In such scenarios, it is preferable to take a directional branch approach or modify the device design to increase the device diameter and minimize the gap distance. Therefore, SESGs, with their adaptability, can accommodate these varying geometries better than BESGs, which might struggle in vessels with significant curvature owing to their inability to conform post‐expansion.

Finally, the difference in anatomical characteristics also contributes to variability in patency rates between renal and visceral arteries for both BSGs. Renal vessels, as indicated in their study and previous reports on the long‐term durability of fenestrated devices, exhibit a higher rate of loss (Graves & Jackson, 2015). The study delves into theoretical considerations regarding why renal branches might be more prone to failure in comparison to visceral branches. Notably, the renal angle displays significant variability among individuals, surpassing that of visceral vessels. This acute angulation might accelerate material fatigue more rapidly using currently available mating stents. Additionally, the considerable respiratory motion experienced by renal arteries, coupled with their smaller size (4–5 mm), might subject the mating stent to increased stress. Furthermore, the increased resistance encountered in perfusing an end organ, as opposed to the low‐resistance systems of the mesenteric and hepatic circulation, could play a role. It is possible that the existing mating stents might necessitate low‐resistance output to maintain patency (Mastracci et al., 2016). Further research is necessary to clarify the extent to which each of these potential explanations influences the findings in this study.

### 3.2 Tortuosity

Another important factor in vascular anatomy is tortuosity (reflecting the curvature of the aorta), which significantly impacts BSG performance. Positioned as versatile workhorses, SESGs are superior to BESGs for vessels with tortuosity and anatomical variations, particularly in cases involving proximal stenosis and luminal irregularities related to atherosclerotic disease (Yoon et al., 2021).

In the experience of Piazza et al. (2021), tortuosity represented a strong factor influencing branch instability, with an eightfold risk when the tortuosity index is >1.15 and a greater effect on stent occlusion (four of the five occlusions were in branches with a tortuosity index of >1.15) (Piazza et al., 2021; Yoon et al., 2021). This outcome, achieved via using SESGs, aligns with other results reported for BESGs (Oderich et al., 2013). Furthermore, aortic endograft and BSG tortuosity have been associated with instability, with tortuous anatomy contributing to increased stent fatigue and subsequent complications. Geometric analysis has underscored the importance of stent characteristics in adapting to vessel tortuosity and ensuring stability after complex EVAR (Finotello et al., 2021). The dynamic challenges encountered during deployment, specifically at the three critical flexion points in the route of the stent (inside the aneurysm sac, at the target artery ostium and at the distal landing transition; Piazza et al., 2021) underscore the need for careful consideration of aortic tortuosity. Larger aneurysm sacs contribute to a more tortuous course for the BSG, impacting its stability and overall performance.

The degree of aortic tortuosity can also affect both the delivery and the deployment of BSGs. In a highly tortuous aorta, challenges during insertion can necessitate increased force, raising the risk of damaging the aortic wall and potentially leading to serious conditions, such as aortic dissection or rupture. Moreover, a highly curved aorta might impede full and symmetrical stent graft expansion, impacting sealing and anchoring and potentially causing endoleaks or migration. In fact, a tortuous aorta has been identified as a predictor for endoleak (Nikol et al., 2020), which can then result in continued aneurysm growth and eventual rupture. In a single‐centre study with 97 TV fenestrations, tortuous vessel anatomy, characterized by a more angled path from the fenestration to the target artery or an upward trajectory of the artery, was associated with higher rates of endoleak 1 year after surgery (Torsello, Herten, Müller, et al., 2020).

Finally, aortic tortuosity can contribute to long‐term stent graft durability issues, because the repeated bending and flexing owing to the aortic curvature can induce fatigue and potential fracture over time, compromising graft integrity and requiring additional interventions. Recently, the risk of BSG tortuosity after complex EVAR was evaluated in a single‐centre study comparing postoperative computed tomography angiography (CTA) polytetrafluoroethylene and BSG failures; the authors concluded that a higher angle of curvature leads to a higher haemodynamic force that results in a higher rate of thrombosis (Xue et al., 2020).

### 3.3 Stent sizing

Stent diameter plays a vital role in the performance and success of BSGs in complex EVAR. In SESGs, the appropriate stent diameter is crucial for ensuring a good fit within the aorta, which minimizes the risk of complications such as endoleaks or stent migration. One that is too large can exert excessive stress on the aortic wall, whereas a stent that is too small might not provide sufficient support. Furthermore, the stent diameter can influence its ability to withstand various forces, including haemodynamic forces, mechanical loads and aortic displacement forces. Therefore, careful consideration of the stent graft diameter is essential for optimizing the procedure and improving patient outcomes.

In addition to the optimal fit, the diameter of the stent graft also influences its flexibility and adaptability to the aortic environment. A larger diameter can offer greater resistance to deformation, but it can also limit the flexibility of the stent graft, making it harder to conform to the natural curves and movements of the aorta. On the contrary, a smaller diameter can provide more flexibility, but it might compromise the resistance of the stent graft to forces, potentially leading to deformation or displacement. Both the curvature of the device and the alteration in diameter around the curved area can contribute to these haemodynamic alterations. In clinical settings, BSGs longer than the original vessels are almost always necessary for convenient cannulation in treating complex aortic aneurysms (Liu et al., 2021).

Although a longer BSG would facilitate more gradual momentum changes and allow blood flow to transition smoothly before reaching the TV (Suess et al., 2016), it would also introduce a curved segment where blood flow could be disrupted, leading to thrombus formation. Haemodynamic parameters are known to be affected by highly curved and tortuous regions, commonly encountered in endovascular management of complex aortic aneurysms. Moreover, the diameter of the stent graft can also affect the haemodynamics within the aorta. One that is too large might disrupt the natural blood flow, leading to turbulent flow or increased blood velocity, which can put the stent graft at risk of displacement. Conversely, a stent graft that is too small might not adequately redirect the blood flow, which could lead to endoleaks or inadequate perfusion of the aortic branches. Furthermore, the diameter of the stent can have a significant impact on the biological response by the body, whereby a larger diameter might induce more inflammation or tissue growth, which could affect the performance and longevity of the stent graft (Liu et al., 2021; Wootton & Ku, 1999). Meanwhile, a smaller diameter might trigger less biological response, but it could also provide insufficient support to the aortic wall, leading to potential complications. Overall, the diameter of the SESG is a critical factor in complex EVAR with BSGs. It requires careful consideration and individualized selection to ensure optimal performance and patient outcome.

### 3.4 Radial force and deployment

Notably, the continual and radially directed expanding force exerted by SESGs post‐deployment has the potential to alter the natural anatomy of the TV, because the stents must apply adequate radial force to ensure a secure seal with the aortic component. It becomes particularly crucial when oversizing vessels, because adequate radial force is essential for proper deployment and conformity of the stent graft to the vessel walls. This property plays a vital role in preventing migration and ensuring the long‐term stability of the stent. The outward expansive force exerted by the SESG before reaching its nominal diameter could explain the ‘delayed expansion’ phenomenon observed in this case (Xodo et al., 2023). This constant radial force helps to maintain vessel patency, especially in dynamic or tortuous vessels, where the stent slowly reaches its intended size, providing long‐term stability but potentially influencing the morphology of the vessel (Xodo et al., 2023). To the best of our knowledge, no prior reports have analysed this occurrence during thoracoabdominal endovascular repair, focusing instead on peripheral arterial procedures.

This is in contrast to the advantage of BESGs, which have more precise positioning during deployment (Yoon et al., 2021). Balloon‐expandable stent grafts exert a high radial force immediately upon deployment, because the balloon inflates the stent to its full size at once. This provides a more rigid and stable structure, particularly useful in calcified or smaller vessels, where a strong seal is required. However, unlike SESGs, BESGs do not continue to apply adaptive force after the initial deployment, which limits their ability to conform to vessel changes but ensures precise initial placement and rapid sealing (Yoon et al., 2021). This makes BESGs advantageous in regions requiring high positional accuracy, and although their rigidity offers initial stability, it might increase the risk of migration or endoleak over time if vessel morphology changes.

The role of relining and its impact on patency warrant further investigation. Previous reports on SESGs have used BMS for relining, demonstrating a high patency rate. The continuous inner forces of a self‐expanding BMS within any bridging stent might enhance the total force applied to the vessel wall, positively influencing patency. However, the introduction of a stent into another stent might lead to a reduction in the diameter of the lumen of the TV (Nana et al., 2023). Balloon‐expandable stent grafts, owing to their high initial radial force and rigidity, might provide similar benefits when relined but are less adaptive to vessel remodelling, necessitating careful sizing to avoid lumen narrowing. Establishing specific criteria, including diameter and angulation thresholds for relining, could provide clarity on its optimal benefit and address questions regarding its role.

Control of deployment can pose challenges in tortuous TVs for both SESGs and BESGs. Longer bridgings characterized by increased tortuosity contribute to heightened resistance in blood flow, elevated lumen shear stress and increased wall tension. Consequently, the risk of occlusion rises owing to potential kinking and turbulence. Self‐expanding BSGs, with their flexible expansion, might better conform to vessel curves, but this flexibility can lead to unpredictable deployment and potential migration in highly tortuous vessels. Balloon‐expandable stent grafts, by providing precise and immediate expansion, might introduce shear stress and turbulence in curved or highly tortuous vessels, leading to long‐term complications. Over the long term, the turbulence induced by a strained path amplifies the occurrence of intimal hyperplasia, ultimately posing a risk of failure for BSGs.

### 3.5 Extrinsic vessel stenosis

Extrinsic stenosis on the coeliac trunk can have a profound impact on the performance of BSGs following complex EVAR. It is a notable complication, with a reported incidence ranging from 12.5%–24%. The main aetiological factors are pancreatitis, tumour invasion or congenital abnormalities (Graves & Jackson, 2015; Yoon et al., 2021). Another common aetiology is compression by the median arcuate ligament, which is a fibrous arch that unites the diaphragmatic crura and can cause compression of the coeliac trunk, leading to stenosis. This compression is most accentuated during end‐expiration, when the two structures move closer together. Stent compression can emerge as a complication, often observed either intraoperatively or during follow‐up, particularly in patients with narrow aortic diameters at the branch level.

Moreover, extrinsic stenosis can impact the physiological flow through TVs and therefore contribute to the failure. Consequently, the amount of blood reaching the areas supplied by the coeliac trunk can be significantly reduced. This can potentially lead to inadequate perfusion. Over time, inadequate perfusion could result in ischaemia, a harmful condition characterized by a lack of oxygen and nutrients in the tissues, which can result in tissue damage or even tissue death. Secondly, the external pressure exerted by the stenosis could potentially deform the stent graft. When the coeliac trunk is narrowed owing to stenosis, it can exert pressure on the stent graft, causing it to change shape or even move from its original location, hence compromising its effectiveness. For example, if the stent is deformed, it might not be able to provide a stable passageway for blood flow, leading to further complications (Oderich, 2017).

Studies have found increased technical difficulties in the performance of BSGs in the coeliac trunk, especially when dealing with extrinsic stenosis (Kim et al., 2022; Torsello, Herten, Müller, et al., 2020). Managing a compressed coeliac artery or one affected by median arcuate ligament compression poses a significant challenge. Although the loss of the coeliac artery rarely leads to significant symptoms, stenting it can be difficult owing to its steep angle. Moreover, ensuring the durability of the stent can be challenging, because it is prone to movement caused by respiratory motion, particularly when aggravated by the median arcuate ligament syndrome. Therefore, understanding the implications of extrinsic stenosis is crucial for optimizing the design, application and long‐term performance of SESGs and BESGs in the context of complex vascular interventions.

In clinical practice, intra‐stenting angioplasty and relining with either uncovered or covered stents are frequently used to reinforce the compressed area (Xodo et al., 2023). The purpose of relining is to straighten the stent, stabilize it, enhance its radial force and mitigate kinking. Nonetheless, the criteria for implementing these supplementary procedures lack standardization, leading to increased costs, a higher frequency of reinterventions and an elevated risk of adverse events (Xodo et al., 2023). Given these potential effects, it is crucial to consider the presence of extrinsic stenosis on the coeliac trunk during the design and application of SESGs. For instance, the BSG could be designed to resist deformation under pressure or measures could be taken during the application of the stent graft to minimize the impact of stenosis on the graft. Studies have shown that covered stents might be advantageous in this context, because they can decrease the rate of stenosis (Yoon et al., 2021). By addressing these issues, the performance and longevity of the stent graft can be optimized, leading to a more favourable clinical profile post‐F/BEVAR (Chait et al., 2022).

Recently, the Nuremberg group reported a novel approach in the treatment of juxta‐renal or supra‐renal AAAs during four‐vessel FEVAR by intentionally choosing not to stent the coeliac fenestration (Ikeda et al., 2009). Despite two reported coeliac artery occlusions, no reintervention was pursued, resulting in an impressive overall TV patency of 97% and no endoleaks. Although unconventional, this strategy provides an alternative for managing complex AAAs in suitable anatomies (Kim et al., 2022).

Conversely, a criticism of the VBX is its lack of connecting bars, potentially making the space between rows, occupied only by polytetrafluoroethyene (PT, more susceptible to constriction and crushing within the graft fenestration. The suggestion of reinforcing the VBX with a BMS could potentially overcome this limitation. However, the systematic use of BMS to reinforce BESGs does not appear to be a definitive solution, especially in long‐term outcomes (Mendes & Oderich, 2017). Regardless of the stent graft used for TVs, the supra‐renal fixation of previous EVAR is considered a challenge and an independent risk factor for type IA endoleaks (Kim et al., 2022).

### 3.6 Respiratory motion

Certain movements within arteries, be it respiratory or pulsatile, have the potential to contribute to premature material degradation, a phenomenon linked to endoleak formation in conventional EVAR settings. Ullery et al. (2016) delved into the impact of geometry and respiratory‐induced deformation on branch vessels following complex EVAR (Marques De Marino et al., 2022), drawing comparisons between branches originating from snorkels and fenestrations. Notably, renal arteries arising from snorkels exhibited significant alterations in end‐stent angle and curvature, particularly during respiration, in contrast to branches originating from fenestrations.

Concerns regarding the influence of curvature and tortuosity on long‐term outcomes extend beyond the aorta. Respiratory‐induced deformation can affect stent positioning and morphology. It is imperative to adopt strategies that optimize stent deployment phases aligned with the respiratory cycle to mitigate potential complications. Given the substantial motion of the aorta during the respiratory and cardiac cycles, the motion of aortic branch arteries during these cycles holds significance (Yoon et al., 2021). Self‐expanding BSGs, with their flexible and adaptive radial expansion, might offer better conformity to vessel movements induced by respiration. Their flexibility allows for dynamic adjustment as the vessels expand and contract during the respiratory and cardiac cycles. This adaptability can reduce the concentration of stress at any single point along the graft, potentially lowering the risk of structural damage such as kinking or fracture. However, this same flexibility can also make SESGs more susceptible to migration in response to repetitive respiratory motion, especially in tortuous vessels. The constant motion can gradually disrupt the initial position of the stent, affecting the stability and seal over time.

Cyclical stress and strain, associated with respiratory and cardiac cycles, not only degrade the attachment of the stent graft but also impact its structure. This can lead to complications such as kinking, fracture and migration. Balloon‐expandable stent grafts are more rigid after deployment. Although this rigidity ensures precise positioning and minimal deformation post‐implantation, BESGs are less capable of conforming to vessel movements. Muhs et al. conducted a study on patients with AAAs using dynamic cine‐computed tomography angiography, which revealed that renal stents restrict the motion of the proximal renal artery, resulting in a 31% decrease in maximal motion (Muhs et al., 2008; Yoon et al., 2021). Moreover, implanting a BeGraft balloon‐expandable covered stent in aortic branch vessels during FEVAR decreased proximal renal artery motion during respiration (Yoon et al., 2021). However, it did not alter the native branch vessel geometry in response to respiratory motion in the coeliac or superior mesenteric arteries. This underscores the importance of developing BESGs that closely replicate native arterial geometry and the cyclic vessel deformation induced by respiration, as supported by additional biomechanical evidence.

When a BEVAR stent graft stabilizes the branch vessel, the motion of the branch artery is transferred along the stent, concentrating the force at its distal end rather than distributing it evenly along the length of the branch artery. Additionally, besides these kinetic forces, there exists a discrepancy in compliance between the BSG and the native branch artery. The combination of kinetic forces and compliance mismatch exposes BSGs to repetitive stress, making them susceptible not only to fracture but also to the development of intimal hyperplasia (Suh et al., 2016; Yoon et al., 2021). The mismatch in compliance between BESGs and the native artery under cyclic motion further exacerbates this issue, because the graft does not distribute the forces evenly along the vessel wall, concentrating strain in specific areas. Strategies involving advanced imaging modalities aid in optimizing stent deployment phases aligned with the respiratory cycle and will contribute to the overall success of complex EVAR.

Consequently, both stent types are susceptible to the cyclical stress and strain caused by respiratory and cardiac motion, which can degrade the attachment of the stent graft to the vessel. Over time, this leads to potential complications such as material fatigue, kinking and, in severe cases, migration. However, BESGs, with their more rigid structure, are more prone to complications arising from compliance mismatch, whereas SESGs can face more challenges related to long‐term positional stability owing to their inherent flexibility.

### 3.7 Haemodynamics and durability

The impact of flow dynamics on failure of BSGs is a complex issue. The hypothesis is that the pinching load on the BSG is related to the remodelling of the aorta and the vessel pulsatility, which might induce plastic deformation (Muhs et al., 2008; Panuccio et al., 2015). Alongside aortic remodelling, the pivotal attribute of radial stiffness (distinctive to BESGs as opposed to the radial compliance and conformability offered by SESGs) plays a crucial role in treating renal and visceral vessels to mitigate complications during follow‐up (Finotello et al., 2021). Nonetheless, a potential drawback of this characteristic is that once BESGs undergo deformation owing to mechanical stress, they are never able to revert spontaneously to their initial configuration. This inherent trait could potentially result in issues such as patency loss, collapse and dislocation (Sugimoto et al., 2016). This is particularly crucial in instances of early bifurcation in a target vessel, where a few millimetres can significantly impact branch vessel preservation and sealing accuracy. The enduring efficacy of various stents used in complex EVAR has been under continuous scrutiny since its introduction. Ideally, these stents should exhibit resistance to kinking, fracture, migration and occlusion. Several widely used stents, including the V12/iCast and the VBX, have displayed fatigue resistance in vitro, presenting promising results for durability (Kim et al., 2022). As additional long‐term data on the durability of complex EVAR stent grafts become available, this will guide the selection of bridging stents and incorporation techniques for visceral vessels.

## 4 HAEMORHEOLOGICAL STATUS: IS IT A MYTH?

### 4.1 Anti‐thrombotic therapy

Reintervention after standard and complex EVAR procedures is common, mainly owing to type II endoleaks, because these might lead to sac expansion over time (D'Oria et al., 2020). The introduction of transarterial embolization to address the endoleak and its associated vessels can be executed using liquid embolic agents such as *n*‐butyl cyanoacrylate and ethylene vinyl alcohol copolymer, thus contributing to therapeutic intervention (Chen & Stavropoulos, 2020). However, primary endoleaks from TVs or BSGs are also frequent after complex EVAR, and a clear characterization of the endoleak source by computed tomography might not possible a substantial proportion of patients (Squizzato et al., 2024). Although most of these endoleaks will resolve spontaneously, during follow‐up some patients might experience a worsened freedom from endoleak‐related reinterventions and require secondary procedures. Further technological advancements, such as the use of intravascular ultrasound (IVUS), might enable better appreciation of intraprocedural issues beyond conventional imaging modalities (Asciutto et al., 2024).

Administration of an antiplatelet agent is advisable following EVAR, according to long‐term therapeutic recommendations. There has also been no proven association between dual antiplatelet therapy and increased rate of bleeding, endoleak or recurrent dissection, and experts generally agree that dual antiplatelet therapy can be useful, particularly when small vessels or longer stents are dealt with (D'Oria, Mezzetto, Silingardi, et al., 2022). However, in vivo evidence suggests that persistent utilization of anticoagulant medications, such as warfarin, presents a potential susceptibility to suboptimal long‐term consequences. This is particularly relevant in terms of BESGs, where the procedure is performed under fluoroscopic visualization, and appropriate anti‐thrombotic therapy should be provided before and after the procedure. The principal end points encompass the absence of regression in the aneurysm sac, sac expansion, risk of endoleak and the rate of reintervention owing to endoleak during the follow‐up period.

### 4.2 Hydration status

The hydration status can influence the postoperative course following bridge stent grafting in complex EVAR. Optimal hydration is crucial for maintaining haemodynamic stability, ensuring adequate renal perfusion and mitigating potential complications associated with the procedure, such as thrombosis or hypoperfusion (Morales et al., 2002). On the contrary, inadequate hydration could compromise tissue perfusion, increase the risk of thrombotic events and adversely affect overall vascular health, hence potentially impacting the success of BSGs. Therefore, vigilant attention to hydration status is essential in the postoperative management of patients undergoing endovascular aortic repair to optimize outcomes and minimize complications during bridge stent grafting. Also, water overload has been shown to be directly related to aortic stiffness (Bia et al., 2015). This, in turn, could impede the smooth advancement and positioning of the BSG, increase risk of embolization associated with a higher burden of atherosclerotic plaque during the procedure, reduce the ability of the aorta to conform to the shape, indirectly affecting the fixation of the BSG, and lastly, increase the risk of postoperative endoleak.

## 5 CONCLUSION

Overall, complex EVAR stands as a transformative therapy in complex AAAs, with BSGs playing a vital role in maintaining the perfusion between the aortic endograft and its TV. The two main types of BSGs are self‐expanding and balloon‐expandable, each with its own distinctive characteristics and mechanism. Physiological factors have been proved to influence the behaviour of both types of BSGs following F/BEVAR, which translates into the clinical profile. Therefore, it is important to consider this interplay between BSG characteristics, vessel physiology and patient haemorheology. Future efforts for development of BSGs should be focused on the direction of closely replicating native arterial geometry and optimizing patient outcomes, until specific custom‐made BSGs emerge on the market.

## AUTHOR CONTRIBUTIONS

Conception or design of the work: Mohamad Bashir, Ian M. Williams, Damian M. Bailey, Mario D'Oria and Matti Jubouri. Acquisition, analysis, or interpretation of data for the work: Thurkga Moothathamby, Matti Jubouri, Tharun Rajasekar, Subham Roy, Maya Alfwaress, Samuel N. S. Ghattas and Samuel S. S. Rezk. Drafting of the work or revising it critically for important intellectual content: All authors. All authors read and approved the final version of this manuscript and hence qualify for authorship, and all those who qualify for authorship are listed. All authors agree to be accountable for all aspects of the work in ensuring that questions related to the accuracy or integrity of any part of the work are appropriately investigated and resolved.

## CONFLICT OF INTEREST

The authors declare no conflicts of interest.
